# A New Variant in the NALCN Channel Is Responsible for Cerebellar Ataxia and Cognitive Impairment

**DOI:** 10.3390/genes16101181

**Published:** 2025-10-11

**Authors:** Rute Luísa Cabrita Pinto, Roberto Fancellu, Tiziana Benzi Markushi, Silvia Viaggi, Barbara Testa, Giuseppina Conteduca, Lane Fitzsimmons, Domenico Coviello, Angela Elvira Covone

**Affiliations:** 1 Laboratory of Human Genetics, Istituto di Ricovero e Cura a Carattere Scientifico Istituto Giannina Gaslini, 16147 Genoa, Italy; ruteluisapinto@gmail.com (R.L.C.P.); silviaviaggi@gaslini.org (S.V.); testabarbara@libero.it (B.T.); giusy_conteduca@alice.it (G.C.); angelacovone@gaslini.org (A.E.C.); 2 Unit of Neurology, Istituto di Ricovero e Cura a Carattere Scientifico Ospedale Policlinico San Martino, 16132 Genoa, Italy; roberto.fancellu@hsanmartino.it (R.F.); tiziana.benzimarkushi@hsanmartino.it (T.B.M.); 3 Clinical Center for Diagnosis of Orphan Patients, Istituto di Ricovero e Cura a Carattere Scientifico Ospedale Policlinico San Martino, 16132 Genoa, Italy; 4 Renaissance School of Medicine, Stony Brook University, 101 Nicolls Road, Stony Brook, NY 11794, USA; lane.fitzsimmons@stonybrookmedicine.edu

**Keywords:** CLIFAHDD syndrome, NALCN, neurodevelopmental disorders, Whole Exome Sequencing, sodium channels

## Abstract

Background/Objectives: CLIFAHDD syndrome (OMIM # 616266) is a rare neurodevelopmental disorder caused by mutations in the NALCN gene. It is characterized by hypotonia, developmental delay, and congenital contractures of the limbs and face. We report a 33-year-old Italian woman with a mild form of CLIFAHDD who exhibited early-onset language difficulties and mild intellectual disability and later developed gait and balance impairments in adulthood. Methods and Results: Whole Exome Sequencing (WES) identified a novel missense variant c.1514A>T; p.(Lys505Met) in the NALCN gene. The allele frequency of this variant is not detected (MAF = 0.0), the variant is classified as likely pathogenic according to ACMG criteria, and predicted to be probably damaging by PolyPhen-2. It affects a critical residue within the second pore-forming domain of the NALCN channel, potentially altering lipid interactions and channel regulation. Sanger sequencing and segregation analysis confirmed the variant to be heterozygous and de novo. Conclusions: The patient’s milder symptoms and later onset, compared to severe pediatric cases, suggest that the clinical spectrum of CLIFAHDD syndrome may be broader than previously recognized. These findings underscore the potential influence of mutation location on disease presentation and severity.

## 1. Introduction

The Congenital Contractures of the Limbs and Face, Hypotonia, and Developmental Delay syndrome (CLIFAHDD, OMIM #616266), first identified by Chong et al. in 2015, is a rare neurodevelopmental disorder [1]. This syndrome was first defined in a cohort of patients who presented with severe atypical phenotypes, including neurological abnormalities and early mortality [1]. These patients were originally thought to have distal arthrogryposis type 2A (DA2A), but they lacked the MYH3 mutation typical of DA2A. Subsequent research identified de novo variants in the NALCN gene as the underlying cause of this distinct condition, known as CLIFAHDD. NALCN encodes a voltage-independent, non-selective cation channel predominantly expressed in the central nervous system [2]. This channel forms the pore of the NALCN channelosome, a multi-protein complex that includes UNC79, UNC80, FAM155, and other proteins [3]. This complex is responsible for the flow of sodium leak currents, which maintain resting potential and regulate neuronal excitability [4]. CLIFAHDD is associated with autosomal dominant variants located within or adjacent to the S5 and S6 transmembrane segments of the NALCN protein, all of which have been reported de novo [5]. The NALCN channel has been identified as a crucial regulator of various physiological processes, including respiratory function, circadian rhythm, motor function, and pain sensitivity [2,6,7,8,9]. Notably, the disruption of the NALCN gene is associated with two distinct conditions: the autosomal dominant CLIFAHDD syndrome and the recessive IHPRF1 (OMIM #615419), which presents with infantile hypotonia, psychomotor retardation, and characteristic facial features.

In this report, we describe the clinical presentation of an Italian patient diagnosed with a mild form of CLIFAHDD, in whom Whole Exome Sequencing (WES) identified a novel de novo missense variant c.1514A>T; p.(Lys505Met) in the NALCN gene.

## 2. Materials and Methods

### 2.1. Family Trio

These findings were obtained within the scope of the diagnostic activities of the Laboratory of Human Genetics. Informed consent was obtained from the family trio for the genetic testing and approved by the Institutional Review Board of the Istituto Giannina Gaslini.

### 2.2. Whole Exome Sequencing and Sanger Sequencing

Genomic DNA from the patient was extracted from peripheral blood using the automated extractor QIAsymphony S (Qiagen, Hilden, Germany). WES was performed on genomic coding regions and exon–intron junctions (5 nucleotides) using the WES_v1: 20,133 genes kit (SOPHiA Genetics, Boston, MA 02215, USA) on Illumina NovaSeq 6000 Sequencing System (RRID: SCR_016387). One hundred and fifty-eight (158) genes of interest (Table S1) were selected based on their associations with the following Human Phenotype Ontology (HPO) clinical terms: cerebellar ataxia (HP_0001251), cerebellar atrophy (HP_0001272), delayed speech and language development (HP_0000750), and intellectual disability (HP_0001249).

Data filtration and interpretation were performed using a variant prioritization pipeline approved for diagnostic analysis—SOPHiA DDM software 5.10.27.2 version—which included Copy Number Variations (CNV) analysis. Sequencing was conducted with an optimal coverage threshold of ≥98% and a minimum target read depth of 20X. Genes marked with an asterisk (*) (See Supplemental Data Table S1) exhibited slightly lower coverage (≥89% and ≤97%) with a 20X target read depth, whereas the NALCN gene achieved full coverage (100%). High-quality variants were filtered based on an alternative allele frequency of ≥30% and a minor allele frequency (MAF) of ≤0.5%, according to the GnomAD database. The Integrative Genomics Viewer (IGV) was used for visualization of sequence data and variant calls.

Pathogenicity of putative germline variants and residue conservation were assessed according to the American College of Medical Genetics and Genomics (ACMG) guidelines [10]. Novel variants were further analyzed through bioinformatic classification using public databases (PolyPhen-2, SIFT, GERP), supported by SOPHiA DDM, Varsome, and ClinVar [11,12,13,14,15]. The proband’s parents served as internal controls to assess variant fraction and coverage thresholds.

Sanger sequencing was conducted on the family trio with BigDye Cycle Sequencing Kit (Applied Biosystems, Waltham, MA, USA) for confirmation and segregation analysis of the identified variant. Table S2 (See Supplemental Data) provides the sequence of the primers used in this analysis.

## 3. Results

### 3.1. Clinical Description

**Figure 1:**
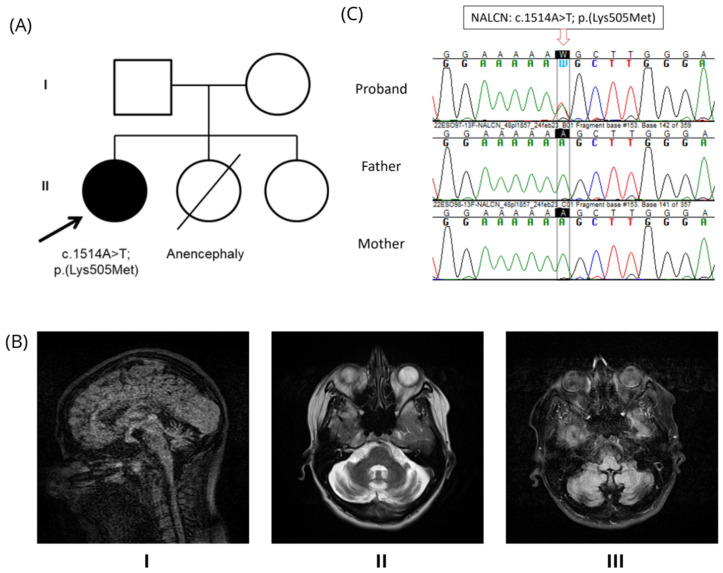
Clinical Description. (**A**) Pedigree. The arrow identifies the proband, and the filled-in circle indicates the presence of the disease. (**B**) Brain magnetic resonance of the proband. (I, midline sagittal section in FLAIR sequence; II, transversal T2 section; III, transversal section in FLAIR sequence) showing global cerebellar atrophy. (**C**) Sanger sequencing results. Chromatograms of the proband, father, and mother are aligned to illustrate the presence or absence of the genetic variant of interest. Abbreviations: FLAIR, Fluid-attenuated inversion recovery.

A 33-year-old woman presented with a history of language difficulties and mild intellectual disability. At age 11, exhibited early signs of language impairment and mild intellectual disability, although the brain MRI was normal. Motor development was largely normal—learned to walk, swim, and ride a bicycle—but had difficulty running. At the age of 17, a comprehensive neuropsychological examination revealed a mild cognitive deficit, with a total IQ of 67 (verbal IQ: 75; performance IQ: 60) on the WAIS scale. The assessment covered multiple cognitive domains, including: language (verbal fluency, token test, Benton and Sartori denomination, PPVT, reading, learning, writing), attention and executive functions (cancellation, Trail Making Test, Stroop test, Elithorn, Labyrinth, London Tower, WCST), memory (forward and backward Corsi and digit spans, word memory), visuo-spatial perception (Ghent-Poppelreuter, Street, Hooper, WAIS subtests), visuo-motor integration and calculating. Performance was below threshold in most areas, with pronounced deficits in language comprehension, selective attention, visuo-spatial processing, and short-term verbal and spatial memory. By age 23, she developed slowly progressive gait and balance impairments with painful cramps in her lower limbs. She did not report sleep disturbances, weight loss, urinary problems, vision or hearing loss, involuntary movements, or epileptic seizures. At the age of 26, the neurological examination revealed: cerebellar gait ataxia without support; inability to walk on toes, heels, or in tandem; moderate limb dysmetria; adiadochokinesia; mild upward ophthalmoparesis; oculomotor apraxia; slurred speech; facial grimaces; inappropriate laughter; and moderate cognitive difficulty in several domains. Her parents are neurologically unaffected and are non-consanguineous. She has a healthy younger sister and another sister with anencephaly who died shortly after birth (Figure 1A). The patient was evaluated using the Scale for the Assessment and Rating of Ataxia (SARA), scoring 16 out of 40. SARA is an internationally validated clinical neurological scale used to assess cerebellar ataxias ([16]). In this case, the score for each item was: gait 3, stance 1, sitting 2, speech 2, finger chase 2.5, nose-finger 0.5, alternating movements 3, and heel-shin 2 [16]. Revaluation of MRI scans revealed a midline vermis area of 641 mm^2^, with a craniocaudal midline diameter of 36 mm, and an anteroposterior midline diameter of 22 mm. (Figure 1B). Brain positron emission tomography revealed cerebellar hypometabolism, whereas the peripheral neurophysiological study was normal. Additional diagnostic tests, including screening for immune-mediated ataxias, antibodies associated with celiac disease, oncological markers, alpha-fetoprotein, vitamin E, and acanthocytes, all yielded normal results.

### 3.2. Genetic Findings

Previous genetic analysis had excluded spinocerebellar ataxia (SCA) related to CAG-triplet expansion, including SCA1, 2, 3, 6, 7, as well as SCA15/16, 17, 28, and 36. The clinical exome sequencing analysis detected a novel germline missense variant c.1514A>T; p.(Lys505Met) in exon 13 of the NALCN (NM_052867.4) gene. The allele frequency of this single-base substitution is not detected, indicating that it is very rare in the population [17]. To predict the impact of the variant in causing disease, public databases were used. PolyPhen-2, a tool that predicts how an amino acid change might affect the structure and function of a human protein, predicted our single nucleotide change as “probably damaging.” The likelihood that the amino acid substitution was tolerated also indicated that the change was deleterious according to SIFT. Furthermore, ACMG scoring by Varsome: 5 points = 5 Pathogenic—0 Benign. The variant fulfilled three pathogenic criteria: PM1 (moderate pathogenic, location in a mutational hot spot/critical domain), PP3 (moderate pathogenic, multiple in silico tools predicting a deleterious effect), and PM2 (supporting: variant not found in gnomAD genomes, variant not found in gnomAD exomes). These criteria correspond to a total of five pathogenic points and no benign points. Conservation analysis indicated that the single nucleotide variant affects an evolutionarily conserved residue, as observed by the Genomic Evolutionary Rate Profiling score (GERP = 5.41) and PhyloP100way (*p* = 7.572). Sanger sequencing and segregation analysis confirmed that our missense variant is in the heterozygous state and is de novo, as neither the father nor the mother is a carrier (Figure 1C).

## 4. Discussion

**Figure 2:**
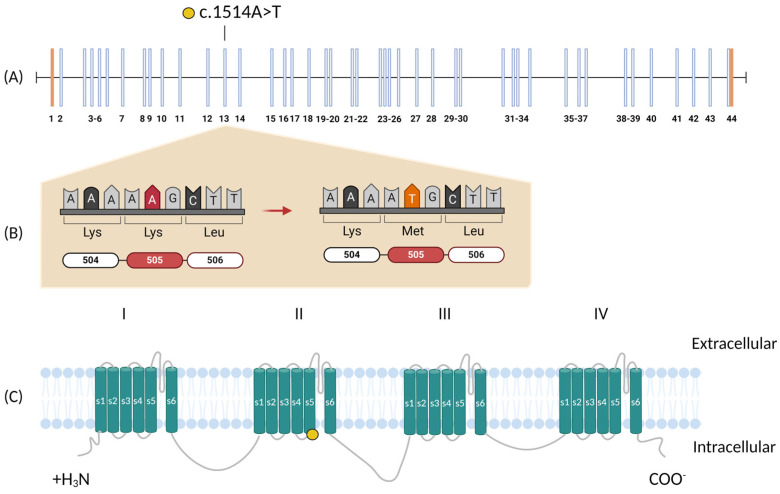
Variant Details. (**A**) Exons of the NALCN gene, including the position of the variant of interest on exon 13; blue bars are coding regions and orange bars are non-coding regions. (**B**) Base change and resulting amino acid change (in red), and (**C**) represents the four pore-forming domains (I−IV) in the NALCN channelsome, with six transmembrane segments each (S1−S6). The location of our variant is indicated by the yellow circle. Created in BioRender. Rute Pinto. (2024).

The patient, a 33-year-old woman, presented with language difficulties and mild intellectual disability, followed by progressive gait and balance impairments in adulthood. Neurological examination revealed motor coordination deficits and moderate cognitive impairment, while neuroimaging demonstrated cerebellar atrophy and hypometabolism. Her clinical presentation aligns with core features of CLIFAHDD syndrome, including motor and cognitive dysfunction. However, the relatively mild symptoms and delayed onset of motor impairments contrast with the more severe, early-onset cases typically reported in the literature. CLIFAHDD is caused by mutations in the NALCN gene, which encodes the NALCN protein, a component of the sodium leak channelosome. Genetic analysis identified a novel de novo missense variant c.1514A>T; p.(Lys505Met) in exon 13 of the NALCN gene, suggested as pathogenic by SIFT, PolyPhen-2, and ACMG criteria. The variant is located in the transmembrane helix segment of the second pore-forming domain (Figure 2) [1]. The second pore-forming domain is markedly different from domains I, III, and IV, potentially due to a unique interaction with lipids. The lysine residue at position 505 is relevant in hydrogen bonding with the phosphate of a lipid molecule: lipid1 [18]. Replacing this positively charged residue with an uncharged methionine would alter such lipid interactions, potentially impacting binding specificity and regulation of the NALCN channel. Mutations in four residues downstream of our variant (p.Leu509Ser) were found to impact NALCN channel function, with increased current density and delayed channel deactivation [1,5]. In a similarly late-onset case, a variant was also identified in protein domain II (p.V597I), but on the opposite side of the channel: the S6 domain transmembrane segment, rather than the S5 domain. Similarly, this patient presented mild symptoms of cerebellar ataxia associated with intellectual disability [19]. The novel variant identified in our patient was submitted on 23 July 2024 to the Leiden Open Variation Database (LOVD) with the individual ID#00452780 (https://databases.lovd.nl/shared/individuals/00452780) and DB-ID: NALCN_000083.

Comparative analysis of previously reported CLIFAHDD cases highlights the phenotypic variability associated with mutations in the NALCN gene. Traditionally, CLIFAHDD manifests with severe symptoms in pediatric patients. However, the milder symptoms and gradual progression observed in our adult patient shed light on genetic variability in the NALCN region. The variability in symptom severity and onset age observed in our patient may reflect the diverse functional consequences of different NALCN gene mutations. These findings suggest that *NALCN* variants influence channel function in diverse ways and that differences in mutation location contribute to the variability in clinical severity observed across CLIFAHDD cases. This underscores the importance of reporting novel mutations, as they enhance our understanding of genotype–phenotype correlations and improve diagnostic and prognostic accuracy across the CLIFAHDD spectrum.

## Data Availability

The original contributions presented in this study are included in the article/Supplementary Materials. Further inquiries can be directed to the corresponding author.

## Supplementary Materials

The following supporting information can be downloaded at: https://www.mdpi.com/article/10.3390/genes16101181/s1. Table S1: Cerebellar ataxia and cognitive impairment gene panel 15 according to Human Phenotype Ontology; Table S2: Primers used for Sanger Sequencing.

## Abbreviations

The following abbreviations are used in this manuscript:

CLIFAHDD	Congenital Contractures of the Limbs and Face, Hypotonia, and Developmental Delay
WES	Whole Exome Sequencing
DA2A	Distal arthrogryposis type 2A
HPO	Human Phenotype Ontology
CNV	Copy Number Variations
IGV	Integrative Genomic Viewer
ACMG	American College of Medical Genetics and Genomics
SARA	Scale of Assessment and Rating of Ataxia
SCA	Spinocerebellar Ataxia
FLAIR	Fluid Attenuated Inversion Recovery
LOVD	Leiden Open Variation Database
MAF	Minor allele Frequency
MRI	Magnetic Resonance Imaging
GnomAD	Genome Aggregation Database
SIFT	Sorting Intolerant From Tolerant
PolyPhen-2	Polymorphism Phenotyping v2
WAIS	Wechsler Adult Intelligence Scale
