# Supplementary material for: A New Variant in the NALCN Channel Is Responsible for Cerebellar Ataxia and Cognitive Impairment
Source: Genes (Basel). 2025 Oct 11;16(10):1181. doi: 10.3390/genes16101181 (PMC12562970; doi:10.3390/genes16101181)
Supplement: Supplementary file 1 [file genes-16-01181-s001.zip › genes-3855989-supplementary.pdf]

Supplemental Data Table S1. Cerebellar ataxia and cognitive impairment gene panel according to Human Phenotype Ontology.

ABCA4	ACAN	ACTN3	AGBL5	ALMS1	ALX4
ANKS6	ANO10	AP3D1	ASAH1	ATN1	ATP2C1
ATXN1	B3GLCT	CACNA1S	CCDC39	CCDC40	CDON
CELSR1	CEP290	CFAP44	CFAP65	CHD3	CISD2
CLDN16	CNTN2	COL11A1	CPAMD8	CPE	CPOX
CSNK1D	DCHS1	DIP2B	DMXL2	DNAH17	DNAH2
DSPP	DYNC2LI1	EGLN1	EMG1	ENTPD1	EPHA2
ESCO2	EVC	EXOSC9	F13A1	FAT2	FBP1
FERMT1	FOXE1	FOXP1	FUK	FYCO1	GALC*
GHR	GIPC3	GJC2	GLA*	GLDN	GM2A
GNAL	GPSM2	H3F3A	HBG1	HLA-DQB1*	HLA-DRB1
HRG	IFT140	IKBKB	IKBKG	IL6ST	IRF6
ITGA3	KCNN3	KDM5B	KERA	KMT2A	KMT2C
KRT14	KRT5	KRT86	LIPA	LRPAP1	MAP3K1
MED12L	MGAT2	MLH3	MMACHC	MUC5B	MUC7
MYH14	MYO5B	MYO6	NALCN	NDUFA13	NDUFC2
NLRP12	NPHP4	NSMCE3	NUTM2B-AS1	PCNT	PDE1C
PEX19	PEX26	PHOX2A*	PIEZO1	PIGQ	PIP5K1C
PKD1	PLA2G5	PLCB4*	PLEC	POT1	POU6F2
PROKR2	QRICH2	RAI1	RBM28	RNF212	ROR1
RP1L1	SCARF2	SCN1B	SCN5A	SETD1B*	SLC12A3
SLC29A3	SLC3A1	SLC44A1	SLC4A4	SLC6A17	SLC6A20
SLCO1B3	SOHLH1	SPINK5	SPRY4	SZT2	TBX4
TENM3	TG	TLK2	TNC	TNFAIP3	TNXB
TONSL	TPP2	TTN	VARS	VCL	VPS13A
VPS13D	WBP2	WDR11	WDR19	WNK4	ZFHX2
ZNF141	ZNF469				

*Target coverage ≥89% and ≤97% with a target read depth 20X.

Supplemental Data Table S2. Primers used for Sanger Sequencing.

Gene-exon-direction	Primer (5’-3’)	Amplicon size (bp)
NALCN - ex13 - F	TATGAATATATTTAAGATTCAGTGTAA	345
NALCN – ex13 - R	AGTGAAATTACTTTGATGTTAATCAT	
